# P-1114. Human Metapneumovirus Outbreak in a Veterans’ Affairs Affiliated Nursing Home

**DOI:** 10.1093/ofid/ofaf695.1309

**Published:** 2026-01-11

**Authors:** Lisa Bailey, Monique Thorne, Debra Noland, Abdullah Khan Zada, Florence M Ford, George Psevdos

**Affiliations:** Northport VAMC, Northport, New York; Northport VAMC, Northport, New York; Northport VAMC, Northport, New York; Stony Brook University Hospital, Stony Brook, NY; Northport VA, Northport, New York; Northport VA Medical Center, Northport, New York

## Abstract

**Background:**

Human metapneumovirus (hMPV) is an enveloped single-stranded RNA virus which can cause acute respiratory tract infections in young, older, and immunocompromised patients. We report a cluster of respiratory illnesses due to hMPV in a Veterans’ Affairs affiliated secure geriatric mental health residential unit.

**Table,:**
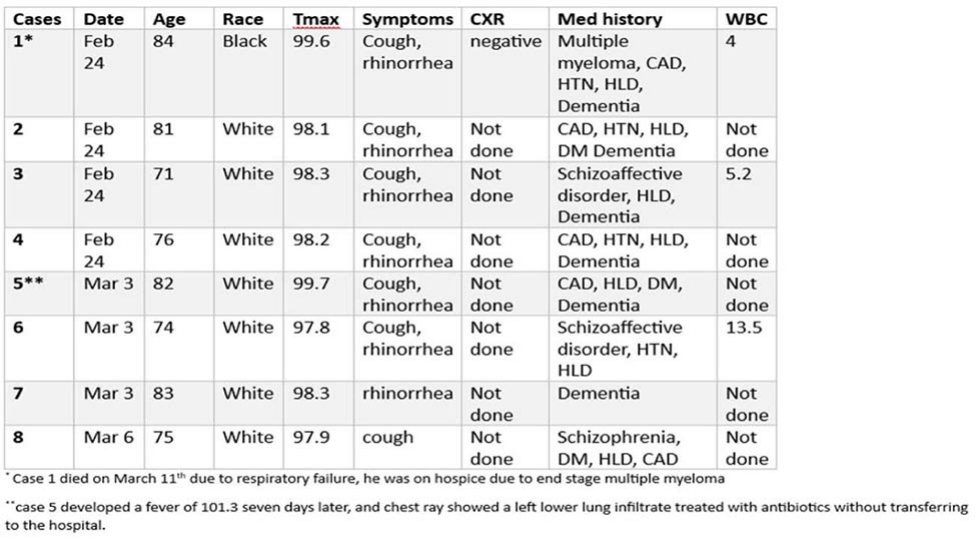
Cases of human metapneumovirus

**Figure:**
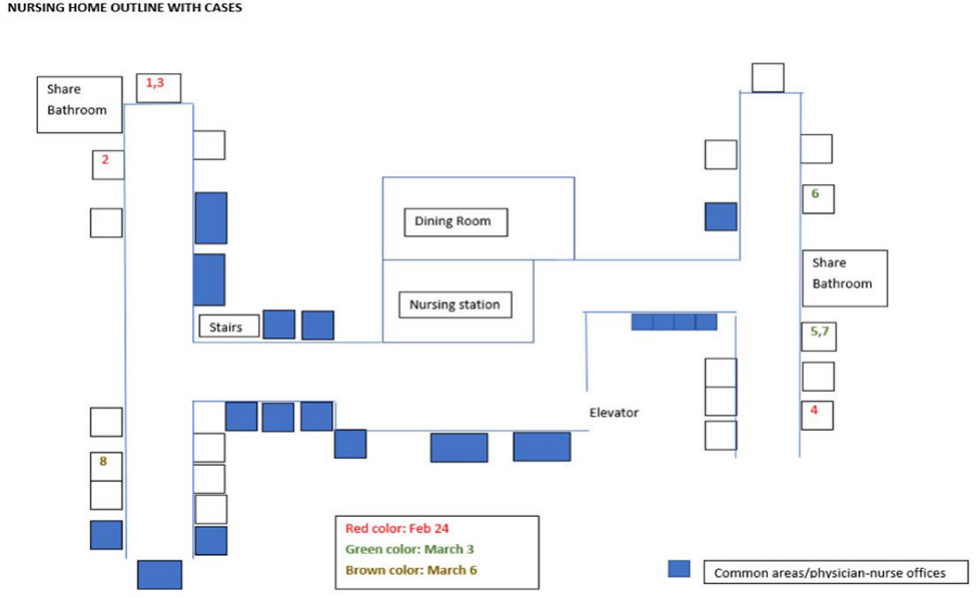
Outline of cases in the nursing home

**Methods:**

A retrospective analysis of an outbreak of hMPV in a 30-bed secure geriatric mental health residential unit in February-March 2025. Cases were confirmed via nasopharyngeal respiratory real-time multiplex PCR panel. Case definition included a resident with new onset respiratory complaints and positive PCR test. Clinical presentation, laboratory data and outcome were reviewed in each case.

**Results:**

The unit houses 25 residents in 17 rooms (single and double room). Eight residents tested positive for hMPV from 2/24 to 3/6/25. All residents were men with ages ranging from 71 to 84 years. On February 24^th^ four cases were identified. Figure 1 depicts the unit and locations of cases. All had mild symptoms and received supportive care, and no one required hospitalization. Case 1 had a visit from his wife a week prior who had congestion and was then diagnosed with COVID-19. Case 1 shares a room with case 3 and a bathroom with case 2. Case 4 is a mobile resident often visiting other residents’ rooms. Case 1 died on hospice on March 11^th^.

Aggressive infection control measures were implemented: halting of admissions; all symptomatic residents were tested; common dining was halted. encouraging residents to remain in their room; teaching respiratory etiquette; enforcing strict hand hygiene and symptomatic residents leaving the residential unit had to wear a mask; terminal cleaning of virus-contaminated surfaces or objects; discouraging floating of staff, directing ill employees to stay home.

**Conclusion:**

We hypothesize that case 1 was infected by his wife, although we don’t have evidence of coinfection (COVID and hMPV). Patients eat together in a communal setting. Cases 1-4 eat at the same table and is likely how others were exposed, hence common dining was halted. hMPV can cause a serious outbreak in residential (nursing) homes. Infection control preventionists can have an instrumental role in rapid recognition of an outbreak and strategically implement targeted measures to halt the outbreak.

**Disclosures:**

All Authors: No reported disclosures

